# The Application of Mohs Micrographic Surgery in the Treatment of Acral Basal Cell Carcinoma: A Report of Two Cases

**DOI:** 10.3390/jcm13226643

**Published:** 2024-11-05

**Authors:** Jakub Żółkiewicz, Laura Banciu, Martyna Sławińska, Mariana Frumosu, Tiberiu Tebeică, Michał Sobjanek, Mihaela Leventer

**Affiliations:** 1 Department of Dermatology, Venereology and Allergology, Faculty of Medicine, Medical University of Gdańsk, 80-210 Gdańsk, Poland; 2 Dr. Leventer Centre, 011216 Bucharest, Romania

**Keywords:** basal cell carcinoma, acral, Mohs micrographic surgery, dermoscopy

## Abstract

**Background/Objectives:** Mohs micrographic surgery (MMS) is a precise skin surgery technique that is particularly useful in the treatment of high-risk skin cancers and tumors located in challenging anatomical areas. **Methods:** we report two cases of basal cell carcinoma located in the acral areas effectively treated using MMS. **Results:** the presented cases demonstrate that MMS is an excellent surgical modality providing outstanding medical, cosmetic, and functional outcomes. Moreover, this study provides another dermoscopic presentation of acral basal cell carcinoma in a patient without the diagnosis of hereditary genetic syndromes associated with an increased risk of skin cancer. **Conclusions:** basal cell carcinoma located on hands and feet, albeit rare, should be included in the differential diagnosis of amelanotic skin tumors in acral areas.

## 1. Introduction

Mohs micrographic surgery (MMS) is a precise surgical technique utilizing histopathological examination for complete evaluation of surgical margins. MMS allows for the complete removal of a tumor while preserving the maximum amount of healthy tissue. Apart from providing the best esthetical and functional outcomes, it is also related to the lowest recurrence rate among skin cancer treatment modalities. The utility of MMS is particularly useful in anatomically sensitive locations such as facial, genital, or acral areas.

Basal cell carcinoma (BCC) is the most frequently encountered skin cancer in Caucasians; however, its acral location has been reported to be extremely rare. A systematic review conducted by Mortada et al. revealed 51 papers on BCC of the hand [1]. Among the reviewed studies, wide local excision was the most frequently chosen treatment method. MMS was performed in 10 out of 51 studies included in the qualitative analysis. In one of the analyzed studies, it has been demonstrated that the average number of stages required to achieve clear margins was >2 and the average final surgical margins after MMS were greater than typically employed in wide local excision for this site [2]. Data on long-term treatment outcomes are limited; however, MMS appears to outperform standard surgical excision, local destructive therapies, and radiotherapy [1]. MMS was successfully utilized in treating acral squamous cell carcinoma (SCC), including cases affecting the nail unit. While it has been shown to be clinically effective, the review authors emphasized that the demanding specimen handling, complex anatomical structure, and distinctive histopathologic presentation mean that MMS in this area should be carried out by skilled Mohs micrographic surgeons [3]. Similar to BCCs, the evidence on MMS in acral SCCs is relatively low and consists primarily of individual case series. MMS has also been used in the treatment of acral melanoma. In a retrospective study of 210 acral melanomas in Korean patients, slow MMS was superior to wide local excision in terms of recurrence, postoperative defect size, and wound healing time [4].

Most of the published acral BCC cases described patients diagnosed with hereditary syndromes associated with non-melanoma skin cancers (NMSCs) (e.g., Bazex-Dupré-Christol, Gorlin-Goltz syndrome, *xeroderma pigmentosum*), whereas spontaneous cases were associated with arsenic exposure, repetitive physical trauma, or burns [5,6]. Herein, we report two cases of basal cell carcinoma located in the acral areas treated using MMS.

## 2. Case Reports

### 2.1. Patient 1

**Figure 1:**
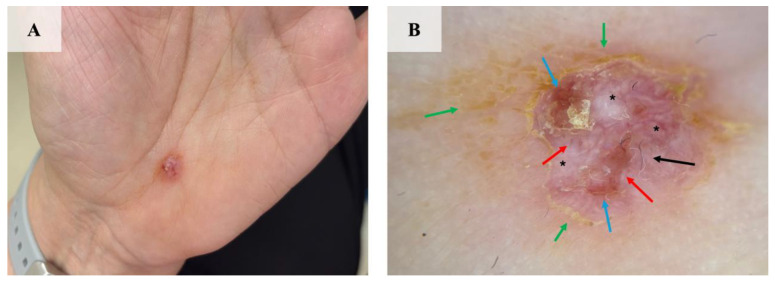
The clinical and dermoscopic presentation of palmar BCC. (**A**) An amelanotic ulcerated nodule located on the left hypothenar measuring 6 × 5 mm. (**B**) Polymorphic vessels (red arrows) overlying milky-red areas (asterisks) were noted in dermoscopy. Moreover, a collarette of yellowish scale (green arrows), multiple small ulcerations (blue arrows), and a “sticky fiber” sign (black arrow) were identified.

A 67-year-old female (phototype II) presented to the tertiary care skin cancer center with a pinkish nodule located on the palm of her left hand (Figure 1A). According to the patient, the lesion was painful and was gradually increasing in size over the previous 5 years. In addition, the patient had a history of hypertension and macular degeneration. The patient did not have any clinical features indicating the diagnosis of hereditary syndromes associated with an increased risk of NMSC. Additionally, the patient had a positive family history of skin cancer; her personal history of skin cancer was negative.

In the clinical examination, a firm amelanotic nodule with a collarette of yellowish scale located on the hypothenar eminence of the left hand was identified. In dermoscopy, polymorphic vessels scattered over a milky-red background were observed. Peripherally, a rim of a yellowish crust spreading out on unaffected skin was noted. Moreover, small foci of ulcerations covered with a serous crust and “sticky fiber” sign were identified (Figure 1B).

**Figure 2:**
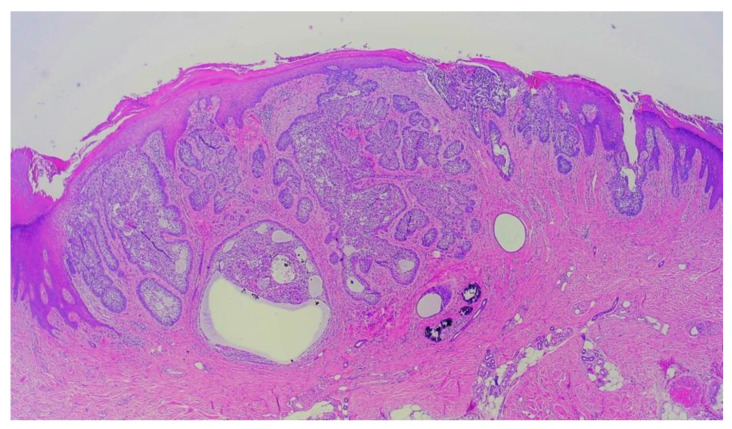
The histopathological presentation of excisional biopsy of nodular BCC of the left palm. Nodular masses of basal neoplastic cells with small hyperchromatic nuclei, nuclear pleomorphisms, scanty cytoplasm, and peripheral palisading of nuclei were identified. (Hematoxylin and eosin staining; magnification, ×4).

**Figure 3:**
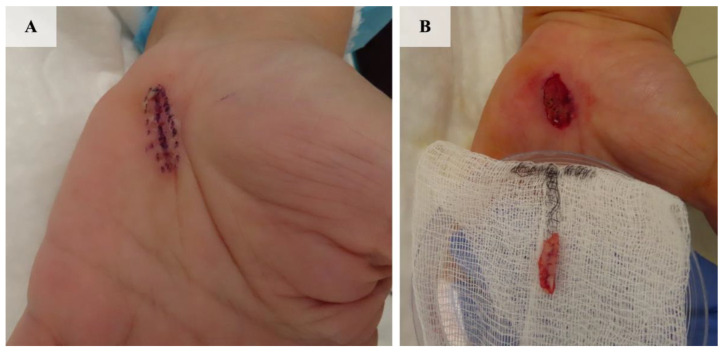
Scar re-excision using MMS. (**A**) Preoperative planning of the lateral excision margins (1 mm). (**B**) Clinical presentation after the first stage of the procedure.

**Figure 4:**
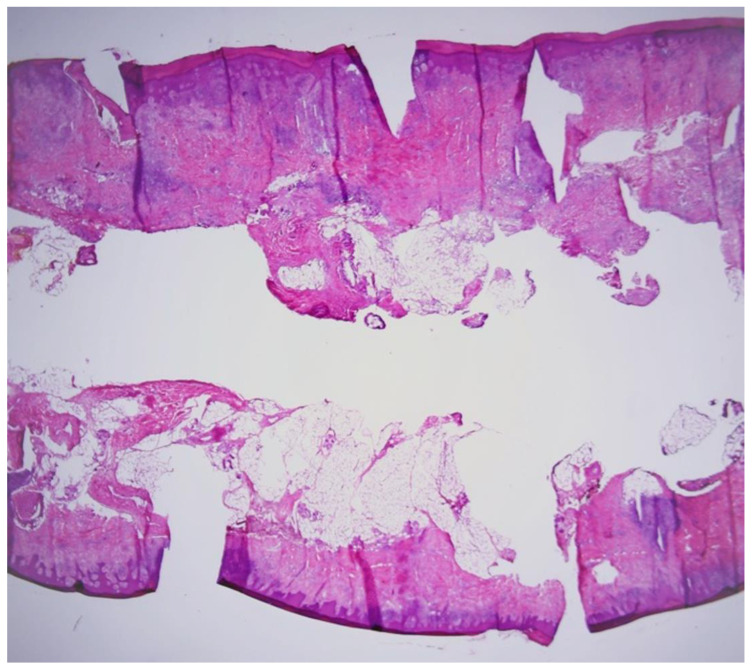
Complete frozen tissue sections revealed no residual cancer. (Hematoxylin and eosin staining; magnification, ×2).

Considering the atypical appearance of the lesion, excision of the lesion was performed, and the histopathological image corresponded to the diagnosis of nodular basal cell carcinoma (Figure 2). Due to the narrow excision margins (up to 1 mm in the deep margins on the examined sections) and the patient’s oncological concern, the scar was subsequently re-excised utilizing MMS (Figure 3A,B). All surgical margins were negative after the first stage of the procedure (Figure 4) and the defect was closed in a linear manner. No postoperative complications were observed.

### 2.2. Patient 2

A 57-year-old male (phototype III) was consulted due to a poorly delineated ulcerated plaque located on the proximal nail fold of the right toe. Over the last year, the patient reported enlargement of the lesion accompanied by recurrent serous exudations. A few years earlier, the patient was diagnosed with onychomycosis of the adjacent nail unit; however, he waived treatment. Concomitantly, the patient was diagnosed with type 2 diabetes, atrial flutter, dyslipidemia, and sleep apnea. No features typical for hereditary syndromes associated with NMSC were detected during clinical assessment, and both his personal and family history of skin cancer were negative.

**Figure 5:**
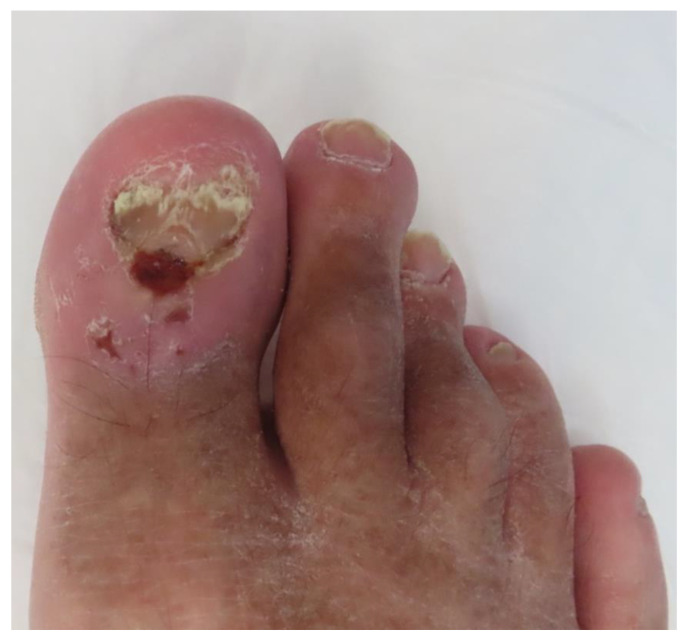
The clinical presentation of infiltrative BCC located on the proximal nail fold of the right toe. A poorly delineated erythematous plaque (10 × 12 mm) infiltrating the eponychium with multiple erosions was noted during physical examination. Concomitantly, dystrophy of adjacent nail plate was present.

An ulcerated plaque infiltrating the eponychium with adjacent small satellite erosions in the proximal aspect of the nail unit was present on the physical examination (Figure 5). Additionally, nail plate dystrophy along with yellow and brown nail plate discoloration were noted.

**Figure 6:**
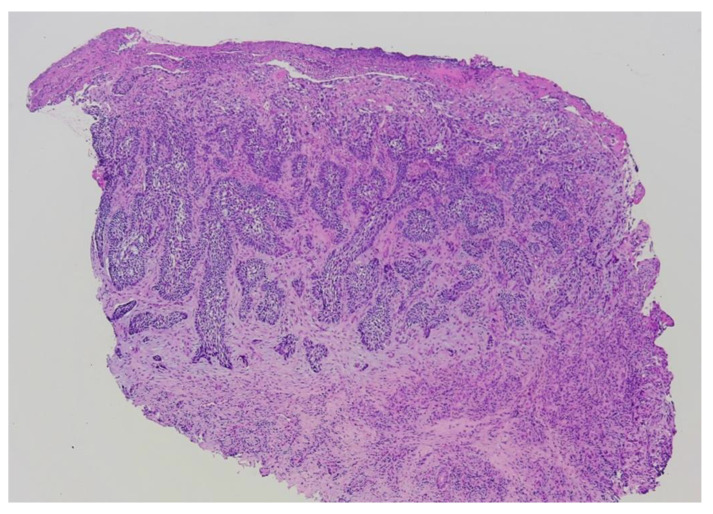
The histopathological presentation of infiltrative BCC of the right toe. A punch biopsy of the ulcerated tumor consisting of tracts of hyperchromatic, pleomorphic basaloid cells emerging from the epidermis. (Hematoxylin and eosin staining; magnification, ×4).

**Figure 7:**
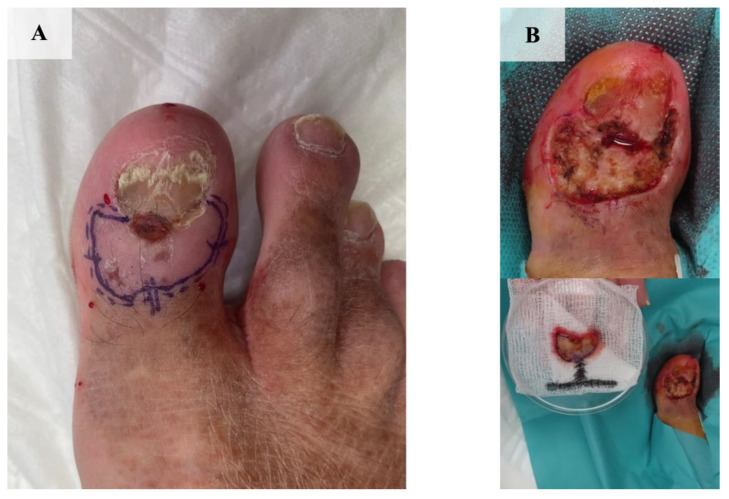
Tumor excision using MMS. (**A**) Preoperative margins of resection. (**B**) Status after first stage of procedure.

**Figure 8:**
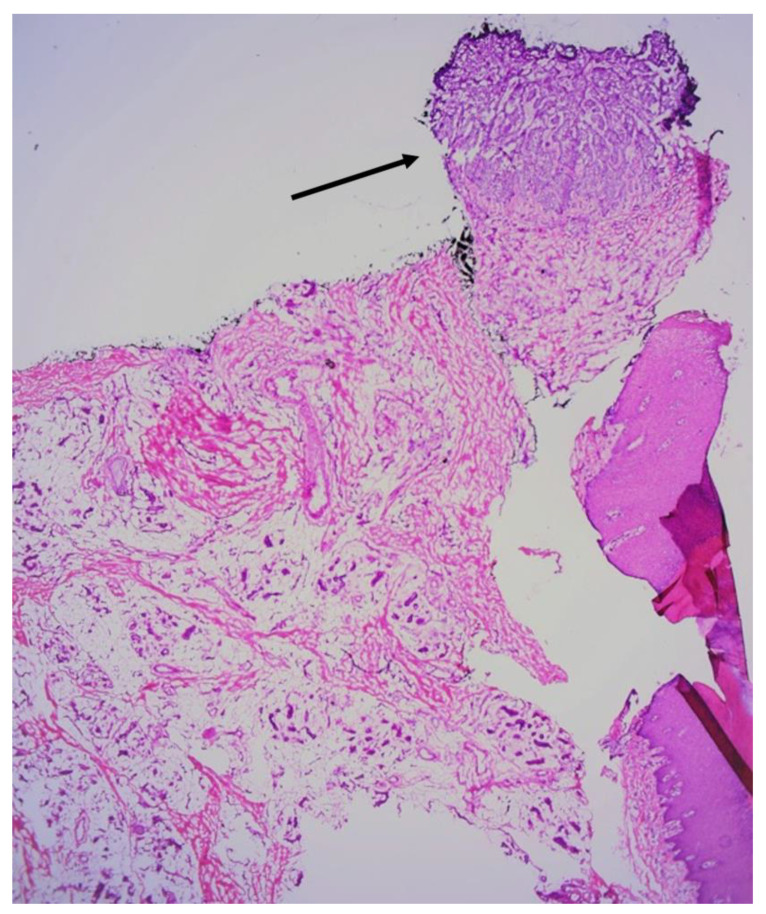
The histopathological image of the frozen tissue sections after the first stage of surgery. Islands of BCC infiltrating the nail matrix were identified at the 6 o’clock position (black arrow). (Hematoxylin and eosin staining; magnification, ×4).

**Figure 9:**
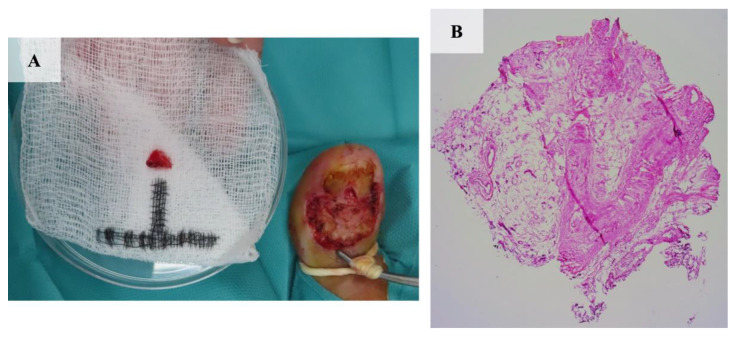
The second stage of MMS. (**A**) A small fragment in the distal part of the defect was excised, and subsequent histopathological examination of frozen sections (**B**) revealed negative resection margins. (Hematoxylin and eosin staining; magnification, ×4).

**Figure 10:**
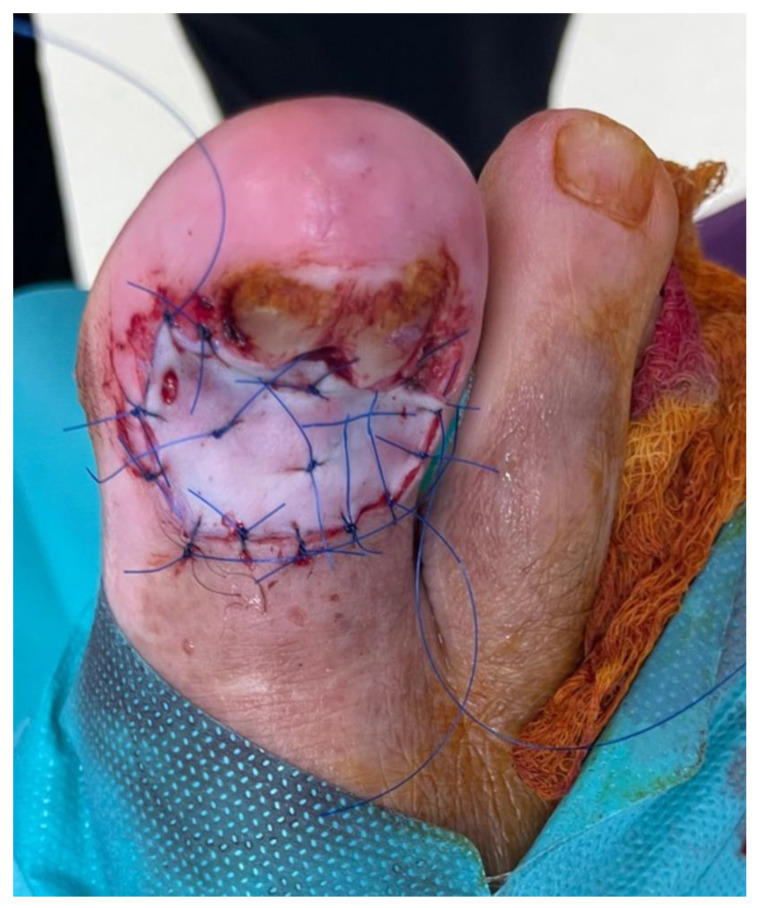
The postoperative result. The defect was closed with a full-thickness skin graft obtained from the thigh.

Due to the untypical clinical presentation of the lesion, a punch biopsy was performed. Based on the histopathological examination, a diagnosis of infiltrative basal cell carcinoma was made (Figure 6). The patient underwent MMS under distal digital block anesthesia (Figure 7A,B). After the first stage of MMS, the margin was positive at the 6 o’clock position (Figure 8). Subsequently, a second stage of surgery was performed, achieving negative margins (Figure 9A,B). The defect was ultimately closed with a full-thickness skin graft obtained from the thigh (Figure 10). The patient was satisfied with the final esthetic outcome of the surgery and reported no postoperative adverse events in the aftermath. No recurrence of the tumor was observed 2 months after surgery.

## 3. Discussion

The etiology of acral BCC remains elusive. It is postulated that BCC originates from keratinocytes of the outer root sheath of hair follicles. This, however, does not explain the occurrence of BCC in proximal nail folds, palms, and soles as they are devoid of hair follicles. One of the hypotheses suggests that acral BCC derives from immature pluripotent epithelial cells analogous to eccrine sweat gland germ cells [7].

Most acral BCC cases have been reported in patients diagnosed with familial skin cancer syndromes such as Gorlin–Goltz syndrome. Some authors believe that palmoplantar pits, which are one of the most prominent features of Gorlin–Goltz syndrome, should be considered BCC in situ [8]. Spontaneous cases of acral BCCs have been reported following trauma, scarring, burns, and exposure to arsenic, among others; however, none of these risk factors were identified in the reported patients. Although BCC is the most common cutaneous malignancy, its occurrence in acral areas is rare. A systematic review performed by Mortada et al. identified 760 cases of BCCs located on hands [1]. An even less frequently encountered location of BCC is the foot, with approximately 50 cases in the literature described so far [6].

The available data on the dermoscopic presentation of acral BCC are scarce [9]. In most cases, blue-grey ovoid nests, milky-red areas, ulcerations, and dotted and serpentine vessels have been described. Interestingly, one dermoscopic hallmark of BCC—arborizing vessels—is rarely encountered in acral BCC cases and we have not detected this in our case either. The structures observed in dermoscopy in our patients fall within the spectrum of previously described cases. Similar to the cases described by Hone et al. and Alonso-Corral et al., we observed yellowish peripheral scaling in both presented cases, which may constitute another clinical clue in the differential diagnosis of acral BCC [10,11].

According to the 2019 European consensus on the diagnosis and treatment of BCC, these tumors can be classified based on the risk of recurrence and based on the treatment difficulty as low/high-risk tumors and easy/difficult-to-treat tumors, respectively. Localization in area H (“mask areas” of face), genital or acral areas is one of the features defining the high risk of a tumor’s recurrence. As stated in the consensus, difficult-to-treat cases encompass locally advanced BCCs or tumors posing particular management problems regardless of the underlying reason [12].

According to the European consensus, the first line of treatment of high-risk BCCs is surgical excision. As outlined in the guidelines, the primary indications for MMS are high-risk tumors, recurrent tumors, and tumors in critical anatomical sites. These recommendations align with those proposed in the position document provided by the European Society for Micrographic Surgery [13].

When dealing with large BCCs, MMS can be combined with neoadjuvant or adjuvant treatment. Topical imiquimod may be used prior to surgery to reduce the extent of the procedure. However, it is important to consider that neoadjuvant therapy with imiquimod may result in the discontinuation of neoplastic nests, which could impede accurate microscopical assessment of resection margins. Imiquimod and photodynamic therapy may also be used postoperatively to treat superficial components of an extensive tumor [12].

When a surgical approach is not feasible, radiotherapy or pharmacological treatment should be considered. Sonic hedgehog inhibitors (SHis) specifically target the hedgehog signaling pathway, which is often abnormally activated in BCCs. Vismodegib and sonidegib are the only SHis which are currently approved for the treatment of locally advanced BCCs. Both drugs have been shown to induce stable disease or even disease remission and are effective either as monotherapy, in combination with radiotherapy, or as a neoadjuvant treatment prior to surgery [14]. Recently, immunotherapy with a PD-1 inhibitor cemiplimab has been introduced as a treatment option for patients with locally advanced or metastatic basal cell carcinoma who have progressed on or are intolerant to SHi. As the overall discontinuation rate of SHi therapy hovers around 90%, PD-1 inhibitors may represent an effective alternative in the therapeutic regimen of advanced BCCs. Although cemiplimab has been registered for the treatment of BCC as a monotherapy both in Europe and United States, ongoing trials show promising results of combining PD-1 inhibitors with other treatment modalities (e.g., relatlimab, ipilimumab, or SHis) [15].

Excisions performed in acral areas are sometimes challenging for a surgeon as, in cases of large defects, complicated reconstructions may be required. High-risk locations, ill-defined borders, and cosmetically and functionally sensitive areas are indications for MMS [13]. Therefore, MMS is an excellent treatment modality for patients diagnosed with acral BCC as it allows for preservation of the maximal amount of healthy tissue whilst ensuring the lowest possible recurrence rate. This is particularly important as acral BCC is associated with a high recurrence rate of 1.28 cases per year [1]. Although MMS is considered a golden standard for treating high-risk BCC, its application in Europe is limited due to the low number of specialized expert centers and both the high amount of time required and financial burden of the procedure.

The diagnosis of acral BCC is made based on histopathological examination, which may be further supported by immunohistochemistry testing for BerEP4. The latter was positive in one of the above-described cases. Although staining for BerEP4 appears to be specific for BCC, negative staining has been reported in cases of acral BCCs [16]. Nevertheless, given the specificity of BerEP4, it may aid differentiating acral BCC from other adnexal tumors. Poroma, adenocarcinoma, squamous cell carcinoma, keratoacanthoma, amelanotic melanoma, and infectious diseases should be included in the differential diagnosis of amelanotic skin tumors located in acral areas.

This study provides additional dermoscopic images of acral BCC. Similar to other papers, the dermoscopic presentation of our cases was not specific for any particular disease. The described cases underline the necessity of performing histopathological verification in cases of peculiar dermoscopic appearance, as acral amelanotic lesions may mimic both benign and malignant entities. In cases of narrow surgical margins after wide local excision, possible further management includes watchful waiting, videodermoscopic follow-up, or scar re-excision. While reflectance confocal microscopy might have served as another alternative, its application in acral areas is restricted due to the limited ability of lasers to penetrate the thick layer of the stratum [17]. Utilizing MMS in the first case ensured complete resection of the lesion and alleviated the patient’s oncological concern while concurrently providing an excellent functional outcome. The second case provided a practical demonstration of MMS’s superiority over standard surgical excision. Wide local excision may entail removal of a large portion of the nail matrix, which could have led to nail unit dystrophy. Due to the infiltrative nature of the tumor, topical therapies and photodynamic therapy were not viable treatment options. By employing MMS only, a narrow part of the nail matrix was removed, yielding favorable oncological, functional, and cosmetic outcomes. Furthermore, this case illustrated that MMS performs exceptionally well in the treatment of poorly delineated lesions in acral areas, as surgical procedures performed on the proximal nail fold convey a risk of subsequent nail unit dystrophy.

MMS should always be considered a treatment option for acral neoplasms as it allows for the precise removal of skin tumors, thus providing higher cure rates and reducing the likelihood of recurrence. MMS allows for the maximum conservation of healthy skin, which is particularly important in acral areas for ensuring the best cosmetic and functional outcomes. Both primary and recurrent BCCs are often poorly delineated and may have complex or extensive roots, which makes their complete removal challenging without MMS. During the MMS procedure, histopathological evaluation of entire tumor margins is ensured, making the MMS technique substantially more effective compared to traditional bread loaf sectioning, which conveys a substantial risk for false negative surgical margins.

## 4. Conclusions

In summary, MMS is an exquisite technique for surgical treatment of acral BCC, providing supreme functional, cosmetic, and therapeutic outcomes. Both presented cases highlight the additive value of MMS in the management of skin tumors in special locations as a method ensuring the best medical and functional outcomes. The reported cases provide another dermoscopic presentation of acral BCC in patients without a diagnosis of hereditary genetic syndromes associated with an increased risk of skin cancer. However, the application of dermoscopy in acral BCC recognition is limited due to the presence of non-characteristic structures such as milky-red areas or dotted vessels which may be observed in other entities such as amelanotic melanoma. Despite its low prevalence in acral areas, BCC should be included in the differential diagnosis of amelanotic lesions located on the hands and feet, including patients without familial skin cancer syndromes.

## Data Availability

The original contributions presented in this study are included in the article. Further inquiries can be directed to the corresponding author.
